# 

**Published:** 1907-04

**Authors:** 


					﻿Vol. XXII.
APRIL, 1907.
No. 10.
T E X A S
C A L
JOURNAL
“RED BACK99
PUBLISHED AT AUSTIN, TEXAS, BY
F. E. DANIEL, M. D.,	-	- Proprietor
PRINTED BY THE VON BOECKMANN-JONES CO.
Subscription, $1.00 a Year, in Advance. «	»	« Single Copies, 10 Cents
Entered at the Pogtoffice at Austin, Texas, as Second-class Mail Matter
				

